# Genome wide transcriptome profiling reveals differential gene expression in secondary metabolite pathway of *Cymbopogon winterianus*

**DOI:** 10.1038/srep21026

**Published:** 2016-02-15

**Authors:** Kamalakshi Devi, Surajit K. Mishra, Jagajjit Sahu, Debashis Panda, Mahendra K. Modi, Priyabrata Sen

**Affiliations:** 1 Department of Agricultural Biotechnology, Assam Agricultural University, Jorhat 785013, Assam, India; 2 DBT-AAU Centre, Department of Agricultural Biotechnology, Assam Agricultural University, Jorhat 785013, Assam, India; 3 Distributed Information Centre, Department of Agricultural Biotechnology, Assam Agricultural University, Jorhat 785013, Assam, India

## Abstract

Advances in transcriptome sequencing provide fast, cost-effective and reliable approach to generate large expression datasets especially suitable for non-model species to identify putative genes, key pathway and regulatory mechanism. Citronella (*Cymbopogon winterianus*) is an aromatic medicinal grass used for anti-tumoral, antibacterial, anti-fungal, antiviral, detoxifying and natural insect repellent properties. Despite of having number of utilities, the genes involved in terpenes biosynthetic pathway is not yet clearly elucidated. The present study is a pioneering attempt to generate an exhaustive molecular information of secondary metabolite pathway and to increase genomic resources in Citronella. Using high-throughput RNA-Seq technology, root and leaf transcriptome was analysed at an unprecedented depth (11.7 Gb). Targeted searches identified majority of the genes associated with metabolic pathway and other natural product pathway *viz*. antibiotics synthesis along with many novel genes. Terpenoid biosynthesis genes comparative expression results were validated for 15 unigenes by RT-PCR and qRT-PCR. Thus the coverage of these transcriptome is comprehensive enough to discover all known genes of major metabolic pathways. This transcriptome dataset can serve as important public information for gene expression, genomics and function genomics studies in Citronella and shall act as a benchmark for future improvement of the crop.

Citronella (*Cymbopogon winterianus* Jowitt) is an important aromatic grass belonging to the Poaceae family with proven therapeutic and medicinal values. Various components of its oil are extensively used as a source of perfumery, soap, cosmetic and flavoring industry throughout the world^1^. Citronella oil is popularly known for its characteristic insect repellent feature^2^. It also possesses many potential bioactive components of great pharmaceutical and medicinal significance which can act as anti-inflammatory, anticancer, antioxidant^3^ andanticonvulsant^4^ agents. The essential oils of Citronella are mostly a blend of monoterpenes and sesqueterpenes containing chiefly limonene, linalool, geraniol, elemol, geranyl acetate, α-bisafalol, citronellol, Citronellal etc^5^. Two common precursors for all these isoprenoid are isopentenyl diphosphate (IPP) and dimethylallyl diphosphate (DMAPP), which are synthesized in two non-related biosynthetic pathways^6^,^7^,^8^,^9^. The initially known one is the cytosolic mevalonate (MVA) pathway where IPP and DMAPP are synthesized from mevalonic acid, the other being plastidial methyl erythritol 4-phosphate (MEP) pathway^10^,^11^,^12^,^13^,^14^,^15^ which uses pyruvate and D-glyceraldehyde 3-phosphate as precursor for IPP and DMAPP synthesis^16^. But to change the influx of various metabolites produced through these pathways, the genes encoding the key enzymes of these pathways along with their regulation must be understood precisely. However, for non-model crops such as *C. winterianus* the paucity of available molecular level information is one of the major bottle necks that impedes their genetic level improvement.

Advances in sequencing technology and bioinformatics tools for annotation of the transcriptome provides a rapid, cost-effective route to develop sequence resources for organisms in which the assembly of a complete genome sequence remains out of reach. Since the last decade, different research groups have exploited these high throughput sequencing technology for better understanding of the molecular level information of various non model plant species such as Chickpea^17^, *Cynara cardunculus*^18^, lentil^19^, *Centella asiatica*^20^, *Thlaspi arvense*^21^, tomato^22^, *Bupleurum chinens*^23^, *Cistus creticus*^24^, *Sesamum indicum L*.^25^, *Cucurbita pepo*^26^, *Camellia sinensis*^27^, *Camillia azalea*^28^, *Andrographis paniculata*^29^, *Asparagus racemosus*^30^ and many more. These studies provided us with much higher molecular level of information as compared to the past. This in turn facilitated in tremendous improvement of these crops through cutting edge molecular biological and genetic engineering techniques. Moreover these data opens up many other field of research in these crops such as molecular marker development, discovery of novel genes, better insight into transcriptional and post transcriptional regulation of important genes, elucidation of metabolic pathway gene which in turn help in metabolic engineering of these pathways etc.

In the present study, we consider an economically important species *C. winterianus*, which is known for its high quality and quantity of essential oils. But, the genes involved in the biosynthesis of the essential oils *i.e.* terpenoids biosynthesis and their molecular regulation are yet to be elucidated. Hardly any sequence data are available for *C. winterianus* in the public databases. Thus a pioneering attempt was made to sequence whole transcriptome of leaf and root tissue of *C. winterianus* so as to gain information on terpenoid synthesis gene with comparative DEG analysis. It is well documented that the leaf and sheath tissues of *C. witerianus* contains high amount of essential oils but root tissue lacks^31^,^32^. Biosynthesis and accumulation of secondary metabolite are often tissue-specific, and related genes of enzymes and regulators also show organ- or tissue-specific expression patterns^29^,^30^. Therefore, we hypothesized that DEG analysis between these two tissues will give a better understanding about the genes involve in secondary metabolite pathway and their regulation. This will provide a step forward for unraveling the major genes behind the terpenes biosynthetic pathway, development of molecular makers as well as further molecular manipulation for better quality and quantity of the essential oils in this high value crop.

## Results and Discussions

### *De novo* assembly and functional annotation of leaf and root tissues

**Figure 1:**
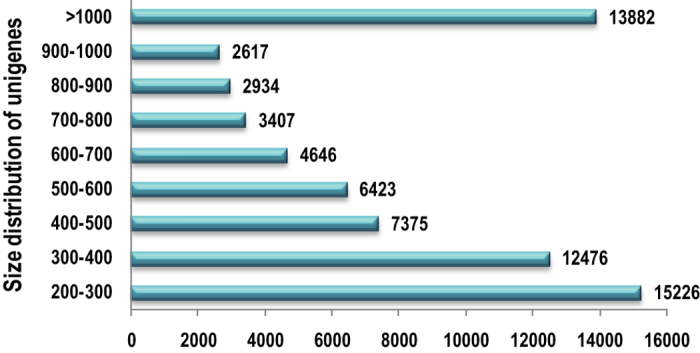
Size distribution of assembled data.

The paired end (PE) library of leaf and root tissue with an average size of 518 bp and 959 bp respectively were subjected to whole transcriptome sequencing. Over 36 million raw reads were generated from both the tissues which approximately amounts to 11.7 Gb data. Both the transcriptome have more than 52% GC contents (Table S1). Average GC content of Citronella transcriptome (53.1%) was well comparable with rice transcriptome (55%)^31^, but it is much higher than Arabidopsis (42.5%), soybean (40.9%) and chickpea (40.3%)^17^. It is well documented that, in the Poaceae family a trend of higher GC content was observed as compared with the few other monocots which may be due to phylogenetic bias^32^. High GC content is also found to be associated with other genomic characteristics i.e., higher gene density (shorter introns)^33^, higher recombination^34^, expression level^35^ and replication timing^36^. The raw data generated in this study were filtered for high quality reads with threshold values higher than or equal to 25. *De novo* assembly of filtered reads were assembled to 68,986 unigenes with size ranging from 200 bp to 1141 bp with an average unigene size of 71 bp. The N50 of both leaf and root transcriptome were recorded to be 590 and 837 respectively, having a high percentage of unigenes size of more than 1000 bp (Fig. 1). The average unigene size was much longer (714 bp) than those reported in previous studies *viz.*, chickpea (428 bp)^17^, Semamum^25^ (629 bp), *Eucalyptus grandis* (247 bp)^37^, *Epimedium sagittatum* (375.9 bp)^38^, *Melitaea cinxia* (197 bp)^39^, *Ipomoea batatas* (202 bp)^40^.

Functional annotation of the leaf and root transcriptome assembled unigenes against NCBI non redundant (nr) protein database GO and KEGG showed highest similarity with *Sorghum bicolor* followed by *Zea mays* and *Setari aitalica* (Figure S1). A total of 92.05% (63,454) unigene sequences showed homology with sequences in the Nr database. The higher percentage of BLAST hits in this study may be attributed to the higher frequency of lengthy sequences of assembled unigenes. As also reported by Parchman *et al.* (2010)^41^, the longer contigs were more likely to have BLAST matches in the protein databases. Data of both the transcriptomes revealed that 94.58% of unigenes over 500 bp in length had BLAST matches. The E-value distribution of the top hits in the Nr database revealed that 56.13% (38,695 unigenes) of the mapped sequences show significant homology (less than 1.0E-50). A total of 5,478 unigenes (7.9%) did not show any homology and can be regarded as novel. Altogether, BLAST searches identified a total of 50,338 unique protein accessions, indicating that in this study the Illumina paried-end sequencing project could generate a substantial fraction of Citronella genes. On the basis of Nr annotation, the Blast2GO program^42^ could annotate a total 27,876 unigenes (16,155 of leaf and 11,721 of root transcriptome) with BLAST matches to known proteins were assigned to gene ontology classes with 56,622 functional terms. Of them molecular function made up the majority (23,830, 42.09%) followed by biological process (20,174, 35.63%) and cellular component (12,618, 22.29%) (Table-S2). The assigned functions of unigenes covered a broad range of GO categories.

### Mapping of KEGG Pathway

A total of 6,724 unigenes were mapped to 135 KEGG pathways. Largest number of unigenes was annotated against purine metabolism, starch and sucrose metabolism, thaimine metabolism, glycolysis pathways etc. The pathway annotation in some other medicinal plants, such as *Uncaria rhynchophylla, Corylus mandshurica* and *Taxus mairei* showed similar type of results. In *Uncaria rhynchophylla* 8,745 unigenes were assigned to 31 KEGG pathways of which 343 belonged to terpenoid synthesis^43^, similarly, in *Corylus mandshurica* a total of 11,056 unigenes were annotated against KEGG database and out of which 29 associated with terpenoid biosynthesis^44^. On the other hand, 11,55ranscripts of *Taxus mairei* were classified to 124 KEGG pathways of which, 69 were assigned to terpenoid 0 tbackbone synthesis^45^. But, as our primary focus is on secondary metabolite associated gene, a comparative analysis of the unigenes mapped in various secondary metabolites associated biosynthetic pathways in both the transcriptome was performed. This analysis revealed that, in the leaf transcriptome a total of 389 unigenes were found to be linked with various secondary metabolite associated pathways *viz.*, terpenoid backbone biosynthesis, sesquiterpenoid and triterpenoid biosynthesis, flavonoid biosynthesis, biosynthesis of terpenoids and steroids. On the contrary, in the root transcriptome only 183 unigenes were annotated against secondary metabolite pathways. Most surprisingly, unigenes annotated in root transcriptome data were primarily associated with drug metabolism, streptomycin biosynthesis, novobiocin biosynthesis and aflatoxin biosynthesis. This comparative analysis clearly indicates that the terpene biosynthesis related genes were mostly expressed in leaf tissue. But, the root of this plant may have different secondary products other than terpenes. This finding adds a new dimension of study in this important aromatic plant albeit from a different perspective.

### Identification of differentially expressed genes (DEGs) in both the tissues

**Figure 2:**
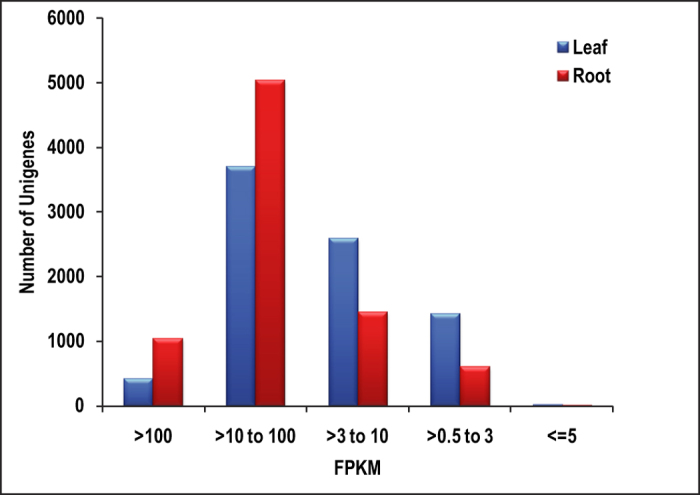
FPKM distribution of DEGs. Dark blue indicates significant up regulation in leaf compared to roots.

**Figure 3:**
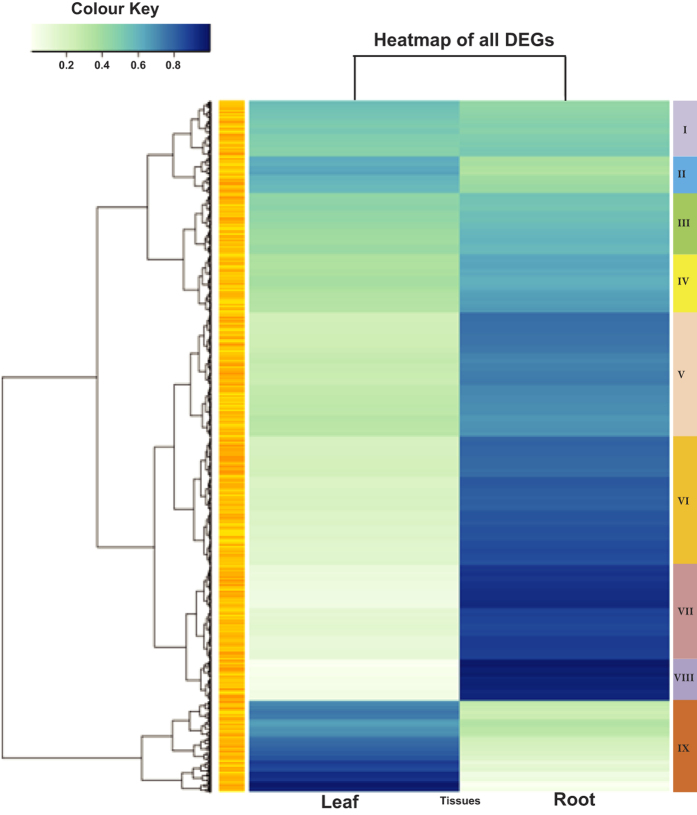
Heat map and complete linkage hierarchical clustering of differential expressed unigenes of leaf and root transcriptome. The various shades of boxes showed similar tendencies of gene expression.

Comparative transcript abundance level revealed significant differential expression of 8,159 unigenes (fold-change ≥2, p ≤ 0.05) between the transcriptome of both the tissues. Although pooled RNA samples of three biological replicates are considered but number of replicate for transctript analysis is one (N = 1), so the differential expression may vary. Levels of expression were represented as log2 ratio of unigenes abundance between leaf and root samples. While working on both the tissues we also observed that a number of transcripts are expressed uniquely in either of the tissues. We found that, 39,582 unigenes were exclusively expressed in either of the tissue, out of which 15,080 (12,341 annotated unigenes and 2739 unannotated) were exclusively expressed in root and 24,502 (21,709 annotated unigenes and 2793 unannotated) in leaf (Supplementary data). To depict the global abundance of gene expression, transcripts were assessed based on FPKM values (Fig. 2), which clearly showed that most of the DEGs fall under FPKM range >10 to 100 followed by >3 to 10. Among them, 652 unigenes were found to be significantly up-regulated while 5,372 unigenes were significantly down-regulated in leaf as compared to root tissue (Log2fold value > 0 for up regulation and Log2fold value < 0 down regulation). The tissue specific DEGs analysis in *Taxus mairei* revealed a total of 6740 differentially expressed genes between the root and leaf libraries with 1,854 genes up-regulated (higher expression in the leaf) and 2,830 genes down-regulated^45^. To gain insight into the differential expression of leaf and root transcripts, complete linkage hierarchical cluster analysis based on FPKM values was performed using R 3.2.0 and bioconductor package (Fig. 3). The heat map of candidate unigenes showed significant differential expression patterns. Of the total 9 clusters, grouped in gene cluster no. III, IV, V, VI, VII, VIII were found to be down-regulated in leaf, while gene in cluster IX was found to be significantly up-regulated in leaf. On the other hand, genes in cluster I and II were found to express almost in equal level with little over expression in leaf as compared to root.

**Figure 4:**
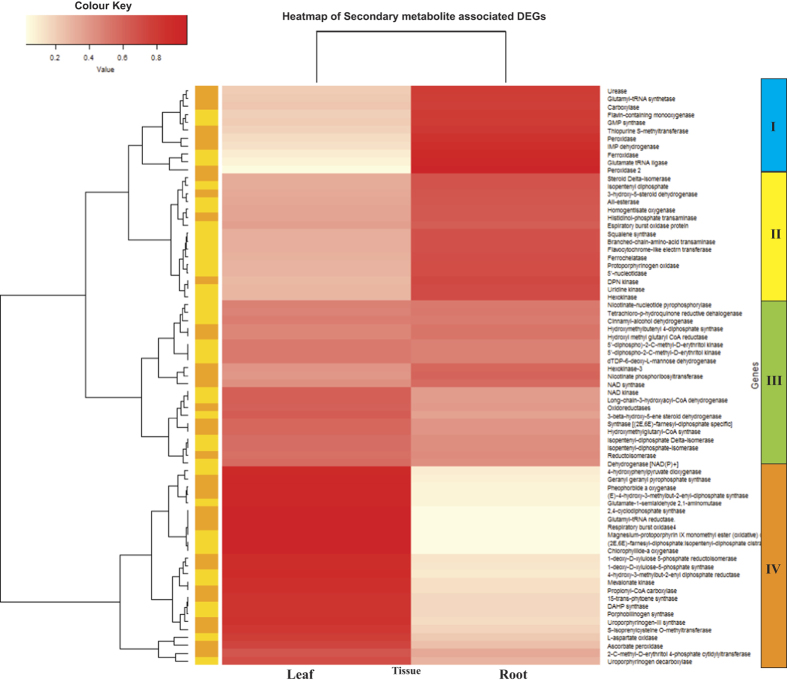
Heat map and complete linkage hierarchical clustering of secondary metabolite associated differential expressed genes of leaf and root transcriptome.

Hierarchical clustering of DEGs associated with biosynthesis of secondary metabolites was prepared to gain a better understanding of differential expression pattern (Fig. 4). The heatmap thus generated grouped the DEGs into four distinct clusters. Genes in Cluster IV were over expressed in leaf tissues, whereas, Cluster I and II genes were highly expressed in root. But, the genes grouped in cluster III showed an intermediary expression pattern in both the tissues. The critical analysis of these clusters revealed, most of the up-regulated genes in leaf tissue belong to MEP, mevalonate and tepenoid backbone synthesis pathways. On the contrary, the genes that were up-regulated in root mostly belong to sesquiterpenoid & triterpenoid biosynthesis, novobiocin biosynthesis, drug metabolism etc. pathways. These results again reinforce the fact that, the essential oils of Citronella are produced in the leaf tissue and may be produced in minimal amount in root. Moreover, the high expression of other secondary metabolite associated genes in the root tissue has added a new dimension. This suggests that this plant may have some other medicinal or economic value that is yet to be explored and thus it opens up future avenue of study.

### K means cluster analysis

**Figure 5:**
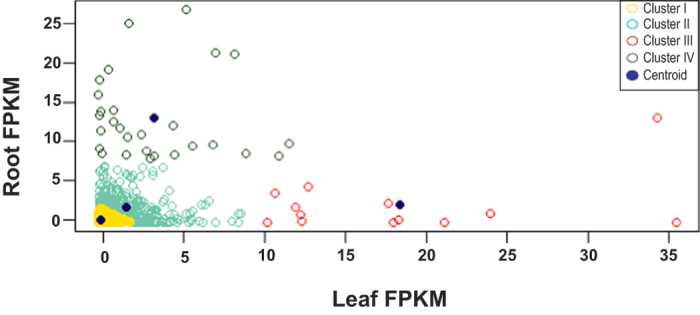
K means cluster of differentially expressed genes.

The appropriate number of clusters to extract, was determined using the within groups sum of square by number of clusters. We looked for an “elbow” bend in the plot similar to a Scree test in factor analysis (Figure S2). From the plot we decided to create four clusters. The cluster plot evidently confirmed that there are a few genes that have high differential expression levels. The genes grouped in the yellow cluster was expressed at a very low level in both samples while in blue was expressing at a higher level compared to yellow. Few of the genes in blue cluster were a little more expressed in leaf than root. The genes in the remaining two clusters i.e., green and red were highly expressed in root and leaf respectively. In the red cluster two genes showed much higher expression level in leaf while their expression in root was negligible (Fig. 5).

### Gene Ontology Enrichment of DEGs

**Figure 6:**
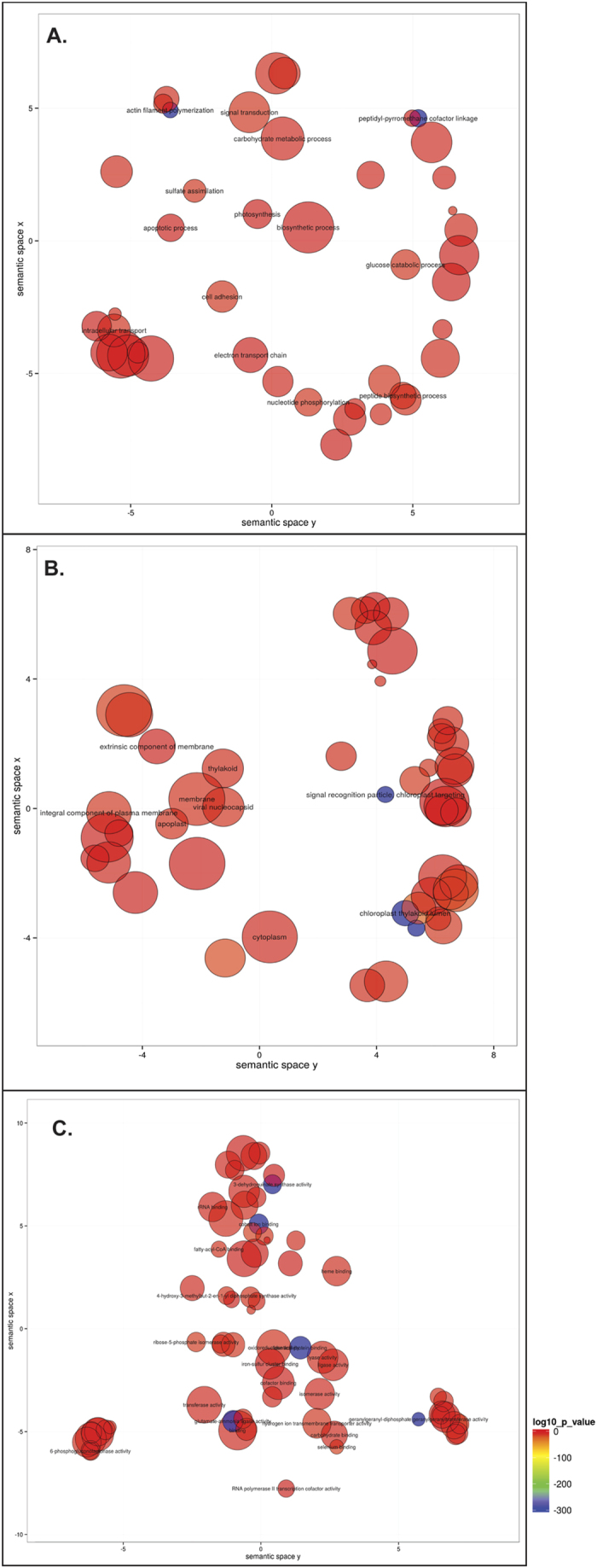
GO enrichment of DEGs. (**A**) Biological Process, (**B**) Cellular Components and (**C**). Molecular Functions.

The GO enrichment in this study provides the statistically significant GO terms associated with the DEGs compared to the background depending upon their frequency. A total of 226 GO terms were mapped against DEGs, whereas 1097 numbers were linked to genes that were not differentially expressed. The frequency of GO terms in DEGs was 785 while in other genes it was 55925. The p-value was calculated for each of the GO terms linked to DEGs using R. 127 GO terms were screened using the p value cut off at 0.05 and finally scattered plot of enriched GOs were visualized in ReviGO. Three components of GO terms i.e. biological process, cellular component and molecular function have been represented in 3 scatter plots (Fig. 6A–C). The scatter plot placed the statistically significant GO terms in the two dimensional plot in such a manner that semantically similar terms gets a nearer coordinates. Among the biological processes, the GO terms linked to signal transduction, photosynthesis, intracellular transport etc. were enriched (Fig. 6A), while GO terms related to chloroplast thylokoid, chloroplast targeting signal recognition particles and exotic component of membranes were enriched among cellular components (Fig. 6B). But, most importantly, in the scattered plot of molecular functions the secondary metabolite associated GO terms, *viz.*, geranylgeranyl diphosphate synthase, geranygeranyl transferase, 3-hydroquinate synthase and 4-hydroxy-3-methylbut-2-en-1-yl diphosphate synthase etc., were significantly enriched (Fig. 6C), which established the fact that these genes have high level of differential expression in leaf.

### Tissue specific differential expression of key enzymes of terpenoid biosynthesis

**Figure 7:**
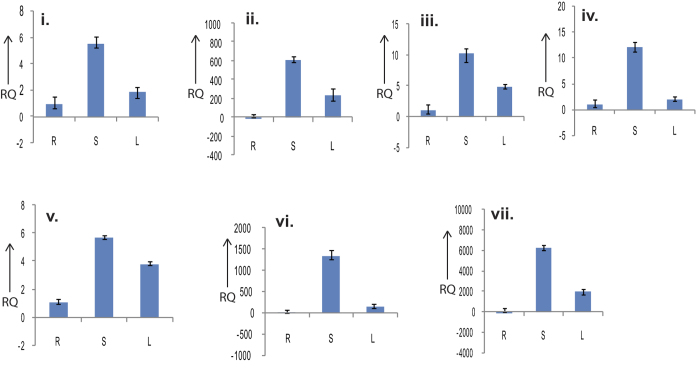
The differential expression study result of MEP pathway genes by qRT PCR in root (R), leaf (L) and leaf sheath (S) of *C. winterianus* using GAPDH as internal control. (**i**) 1-deoxy-D-xylulose 5-phosphate synthase, (**ii**) 1-deoxy-D-xylulose5-phosphate reductoisomerase, (**iii**) 4-phosphate cytidylyl transferase, (**iv**) 5′-diphospho-2-C-methyl-D-erythritol kinase, (**v**) 2,4-cyclodiphosphate synthase, (**vi**) (E)-4-hydroxy-3-methylbut-2-enyl-diphosphate synthase, (**vii**) 4-hydroxy-3-methylbut-2-enyl diphosphate reductase and viii. GAPDH.

Fifteen terpenoid biosynthesis genes were selected for tissue specific expression profiling by Reverse Transcriptase (RT) and quantitative Real Time (qRT) PCR. Accordingly gene specific primers were designed (Table S3). All the seven genes from the plastidal methyl erithritol phosphate (MEP) pathway *viz.*,1-deoxy-D-xylulose 5-phosphate synthase (DXS), 1-deoxy-D-xylulose5-phosphate reductoisomerase (DXR), 4-phosphate cytidylyl transferase (CMS), 5′-diphospho-2-C-methyl-D-erythritol kinase (CMK), 2,4-cyclodiphosphate synthase (MCS), (E)-4-hydroxy-3-methylbut-2-enyl-diphosphate synthase (HDS) and 4-hydroxy-3-methylbut-2-enyl diphosphate reductase (HDR), were selected for validation. We could observe a distinct differential expression pattern of these genes with highest expression in leaf sheath followed by leaf and lowest in root (Fig. 7). Both RT (Figure S3) and qRT PCR result highly corroborate with one another confirming high expression of MEP pathway genes in leaf sheath followed by leaves. MEP pathway genes are highly expressed in aerial parts and plastidal in nature^10^,^11^,^12^,^13^,^14^,^15^,^16^. The present study re-establish the fact that, these genes code for plastids specific enzyme. Although, in number of such studies on expression profiling of MEP pathway genes in different plants species, reported higher level of expression found in leaf tissues than other tissues tested^21^. But, it can be suggested that high level of mRNA is not enough to reflect the level of enzyme activity in a particular cell type, as mRNA or protein itself is transportable to other site of action. This result might be an indication that the expression of MEP pathway genes is high in plastids abundant in phloem and other cells of sheath tissue. Similar results have also been reported in northern blot and immunolocalization experiment conducted in *Catharanthus roseus* for MEP pathway genes, which revealed that, internal phloem plays a key role in formation of precursors and decoration of monoterpenoids^33^. Thus, several mechanisms of transport must exist to deliver isoprenoid intermediates out of the phloem parenchyma plastids and to other compartments and/or cells where they will be further metabolised or stocked^33^.

**Figure 8:**
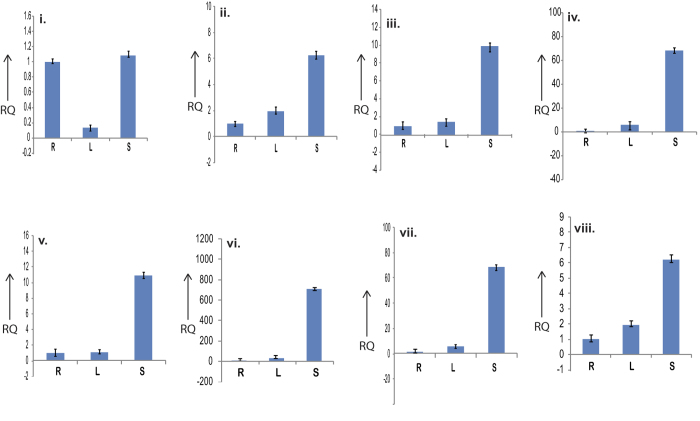
Differential expression study result of four Mevalonate pathway genes by qRT PCR in root (R), leaf (L) and leaf sheath (S) tissue of *C. winterianus* using GAPDH as internal control (**i**) HMG-CoA reductase, (**ii**) Mevalonate kinase, (**iii**) Diphosphomevalonate decarboxylase, (**iv**) hydroxymethylglutaryl-CoA synthase, (**v**) Geranylgeranyl pyrophosphate synthase, (**vi**) (**2E**,**6E**)-farnesyl-diphosphate synthase, (**vii**) S-isoprenyl cysteine O-methyl transferase, (**viii**) Isopentenyl-diphosphate Delta-isomerase and ix. GAPDH.

Four genes were analysed from the mevalonate pathway *viz.*, hydroxyl methyl glutaryl CoA reductase (HMGR), mevalonate kinase, diphospho mevalonate decarboxylase and hydroxy methyl glutaryl-CoA synthase (Fig. 8i–iv). Except HMGR, all other genes showed highest expression in leaf sheath followed by leaf and least in root tissue. This result again indicated higher production of these compounds in sheath tissue and final metabolites may be transported to leaf tissue subsequently. Similar type of result was also reported in *Picrorhiza kurroa* by Pandit *et al.*^46^, where they reported higher expression of these genes in stolon followed by leaf and least in root tissue. On the contrary, both the RT and qRT PCR result showed that HMGR is expressing almost at equal level in both leaf sheath and root tissue (Fig. 8i). HMGR being a cytosolic enzyme and first rate limiting enzyme of MVA pathway is responsible for production of number of diverse products such as squalen, ubiquinones, sterols, terpenes etc.^47^, which may be expressing equally in both underground and above ground part of this plant. Therefore, expression of HMGR can be equal in both root and leaf sheath tissue, but the flux of intermediary product may divert in different pathways in different tissues to produce varying metabolites.

Finally, four more genes from the terpenoid backbone biosynthesis pathway were analysed *viz.*: geranylgeranyl pyrophosphate synthase, (2E,6E)-farnesyl-diphosphate synthase, S-isoprenylcysteine O-methyltransferase, isopentenyl-diphosphate Delta-isomerase. All the enzymes showed similar but distinct pattern of differential expression with highest expression in leaf sheath tissue followed by leaf and least in root (Fig. 8v–viii). These are the downstream genes of MEP Pathway compartmentalized in plastids^48^,^49^. Therefore, it is quite predictable that, as the upstream enzymes were expressing at higher level in sheath tissue so the substrates for these four downstream enzymes will be more abundant in the same tissue. Thus, these enzymes were also expressing at higher level in sheath tissue. The RT-PCR results (Figure S4) for all these genes exactly corroborate with the qRT-PCR result.

Finally, from the present study, an extensive transcriptome dataset has been generated from deep sequencing of *Cymbopogon winterianus*. The coverage of the transcriptome data is exhaustive enough to discover all known genes involved in the terpenoid pathway. Thus the present study constitutes the largest report of transcriptome based gene resources data on *C. winterianus*. This shall act as benchmark for multi-faceted future improvement of the plant through discovery of new genes, marker assisted breeding in this plant and also terpenes producing other medicinal plants.

## Materials and Methods

### Plant material and total RNA isolation

A promising high yielding local variety of *Cymbopogon winterianus*, Jorlab-1, developed by North East Institute of Science and Technology (CSIR), Jorhat, India, was grown in the Instructional cum Research (ICR) farm of Assam Agricultural University, Jorhat, India. This variety is known for its high content of essential oil and high recovery rate. The variety flowers during the months of December to February. The leaf and root tissues were collected 4 months after plantation in three biological replicates for each tissue. Total RNA was isolated separately for each replicate using 100 mg tissues. The tissues were homogenized in liquid nitrogen to a fine powder and total RNA was isolated by using RaFlex Plant RNA miniprep kit (Genei, India) as per manufacturer’s instruction. The quality of isolated RNA was checked in 1% denaturing agarose gel for the presence of intact 28S and 18S bands. The quality and quantity of isolated total RNA was checked using Nanodrop-1000 spectrophotometer (Thermo Scientific, USA) using 1 μl of the sample. Then replicates were pooled together for preparation of leaf and root cDNA libraries.

### Sequencing and *de novo* assembly

The pair-end cDNA sequencing library was prepared using Illumina TruSeq RNA sample preparation v2 kit (Illumina, USA) as per manufacturer’s instruction. Quantification and quality assessment of resulting libraries were performed on 2100 bioanalyzer (Agilent Technologies) using dsDNA High Sensitivity DNA chip as per manufacturer’s instructions. 10 pmol of library was loaded into the reagent Cartridge of MiSeq (for leaf) and NextSeq (for root). Paired-End sequencing allows the template fragments to be sequenced in both the forward and reverse directions.

The raw data generated was filtered using Trimmomatic v0.30. Parameters considered for filtration were adapter trimming, cutting the average quality read (threshold of 25), removal of bases of the start of a read and the end of a read (Score <25) along with dropping off the reads below 50 bp length. The GC content analysis provides insight into various aspects related to genome of an organism, including evolution, gene structure, thermo-stability and gene regulation was calculated using the program CodonW1.4.2 (http://codonw.sourceforge.net/) and FastQC tool.

### Functional annotation of the unigenes

The high quality data of Citronella leaf and root samples were assembled using CLC genomics workbench v.6.0 on default parameters with mismatch cost 2, insertion and deletion cost 3. The unigenes were validated by mapping all the HQ data on the assembled unigenes. The functional annotation was performed using the unigenes of the samples by aligning those unigenes to non-redundant protein database of NCBI using BLASTX.

Gene Ontology (GO) terms were assigned to the assembled unigenes (consensuses and singletons longer than 200 bp) through Blast2GO (B2G) package^50^. The GO analysis helps us in specifying all the annotated sequences comprising of GO functional group such as Biological Process, Molecular Function and Cellular Component.

Mapping of unigenes to the biological pathways was done by aligning the unigene sequence against the Kyoto Encyclopaedia of Genes and Genome (KEGG) database using BLAST2GO software. Enzyme commission (EC) numbers were assigned to unique sequences after BLASTX searches with an E-value cut-off of <1.0e^5^ against the KEGG database. The sequences were assigned to specific biochemical pathways according to the corresponding EC distribution in the KEGG database.

### Differential gene expression and K means cluster

Differentially expressed genes identified in Citronella leaf and root tissues were analyzed using Multiple Experiment Viewer (MEV v4.8.1) software. Levels of expression were represented in log2 ratio of unigenes abundance between Citronella leaf and root samples. Clustered heatmap analysis was performed based on the FPKM (Fragment per kilo base of exon model per million mapped reads) values using ‘annheatmap2’(R Bioconductor). The heatmap was constructed using log-transformed and normalized value of unigenes based on average linkage method.

For k-means cluster analysis, the number of cluster was determined using the within groups sum of square by number of clusters. We looked for an “elbow” bend in the plot similar to a Scree test in factor analysis (Figure S5).

### GO enrichment of DEGs

The DEGs associated GO terms were enriched with respect to the GO terms associated to the genes not expressed differentially. A hypergeometric test equivalent to one-tailed Fisher’s exact test was performed to find the over-represented GO terms in the DEGs. P values was calculated based on χ^2^ score of FPKM value of each DEG and finally computed from the chi-square distribution curve. The P value cut off was put at p < 0.05 and all the GO terms qualifying this parameter were visualized using ReviGO^51^.

### Tissue specific differential expression analysis

The differential expression of the 15 secondary metabolite associated genes in three different tissues of Citronella (leaf sheath, leaf, root) were tested by semi-quantitative RT PCR and quantitative real time PCR. The primers were designed by primer3 (http://primer3.ut.ee/) with GC% of 60–65% and melting temperature ranging from 60–65 °C. The first strand cDNA^52^,^53^,^54^,^55^,^56^ was synthesized by using PrimeScript TM 1^st^ strand cDNA synthesis kit (Clontech Takara, USA) as per manufacturer’s instruction. The 10 μl reaction mixtures contained 125 ng first strand cDNA with 1X Taq polymerase buffer (Genei, India), 1U Taq polymerase (Genei, India) 10 pmol both forward and reverse primers (Sigma Aldrich, USA) and 5 mmol dNTP mixure (Invitrogen, CA, USA). This was subjected to semi-quantitative RT PCR in GeneAmp Thermo Cycler (Applied Biosystem, USA). The amplification condition were 5 min at 94 °C, followed by 35 cycles of 1 min at 94 °C 1 min at 67.5 °C 1 min at 72 °C and final extension was done for 10 min at 72 °C. The amplified products were visualized in 2% agarose gel stained with EtBr. In this study rice glyceraldehyde-3-phosphate dehydrogenase (GAPDH) gene was used as reference. The relative expression of MEP pathway genes in the same three tissues were checked with quantitative real time PCR by ∆∆CT method^57^ on StepOnePlus Real-Time PCR System (Applied Biosystem, USA) using the SuperScript III Platinum SYBR Green One Step qRT-PCR with ROX Kit (invitrogen, CA, USA) according to manufacturer’s instruction. For each sample three technical replicates were taken based on which the error bars were calculated. PCR amplification was performed under the following conditions: 95 °C for 10 minute, followed by 40 cycles at 95 °C for 15 second and at 60 °C for 1 minute finally melting at 95 °C for 15, 60 °C for 1 minute. The gene expressions were normalized against an internal reference gene, GAPDH and root tissue was arbitrarily chosen to be the calibrator of tissue gene expression.

## Additional Information

**How to cite this article**: Devi, K. *et al.* Genome wide transcriptome profiling reveals differential gene expression in secondary metabolite pathway of *Cymbopogon winterianus*. *Sci. Rep.* **6**, 21026; doi: 10.1038/srep21026 (2016).

## Supplementary Material

Supplementary Information

Supplementary Dataset 1
