# Supplementary material for: Genome wide transcriptome profiling reveals differential gene expression in secondary metabolite pathway of Cymbopogon winterianus
Source: Sci Rep. 2016 Feb 15;6:21026. doi: 10.1038/srep21026 (PMC4753472; doi:10.1038/srep21026)
Supplement: Supplementary Information [file srep21026-s1.doc]

**Genome wide transcriptome profiling reveals differential gene expression in secondary metabolite pathway of *Cymbopogon winterianus***

Kamalakshi Devi^1^, Surajit K Mishra^2^, Jagajjit Sahu^3^, Debashis Panda^3^, Mahendra K Modi^1,3^, and Priyabrata Sen^1^*

*1Department of Agricultural Biotechnology, Assam Agricultural University, Jorhat-785013, Assam, India*

*2DBT-AAU Centre, Department of Agricultural Biotechnology, Assam Agricultural University, Jorhat-785013, Assam, India*

*3Distributed Information Centre, Department of Agricultural Biotechnology, Assam Agricultural University, Jorhat-785013, Assam, India*

*E-mail:* [*pbsen14@yahoo.co.in*](mailto:pbsen14@yahoo.co.in)

Table S1. Comparison of De novo transcriptome assembly

| **Description** | **Leaf** | **Root** |
| --- | --- | --- |
| Number of unigene | 40823 | 28163 |
| Total unigene size (bp) | 24075811 | 23554959 |
| Average unigene size (bp) | 590 | 837 |
| Unigene N50 (bp) | 843 | 996 |
| Max unigene size (bp) | 7479 | 11412 |
| Min unigene size (bp) | 200 | 297 |

**Table S2,**  GO distribution of citronella unigenes

| **GO Terms** | **Leaf** | **Root** | **Total** |
| --- | --- | --- | --- |
| **Biological processes** | 10,872 | 9,302 | **20,174** |
| **Molecular functions** | 14,184 | 9,645 | **23,829** |
| **Cellular components** | 5,915 | 6,703 | **12,618** |
| **Total** | **30,971** | **25,650** | **56,621** |

**Table S3**. List of gene specific primers

| ­­­­­­­Sl No | Gene name | EC number | Source unigene | Primer | Length | Tm | GC% | Primer Sequences 5’---3’ |
| --- | --- | --- | --- | --- | --- | --- | --- | --- |
| 1. 1 | 2,4-cyclodiphosphate synthase | ec, 4.6.1.12 | unigene_21208 | F | 22 | 65.00 | 59.09 | 5’-AAGAGTCGGCACCCTTCCAACG-3’ |
|  |  |  |  | R | 21 | 63.74 | 57.14 | 5’-GCTCATCATCGGCGGCATCAA-3’ |
| 1. 2 | Diphosphomevalonate decarboxylase | ec, 4.1.1.33 | unigene_2894 | F | 20 | 65.73 | 60.00 | 5’-ACGCCCACCAACATTGCGGT-3’ |
|  |  |  |  | R | 21 | 64.31 | 57.14 | 5’-TCTCCTTGCCGTTGAGCCACA-3’ |
| 1. 3 | 4-phosphate cytidylyltransferase | ec, 2.7.1.148 | unigene_3851 | F | 22 | 64.17 | 54.55 | 5’-TGCACGCCCAGGAAAAGAGAGA-3’ |
|  |  |  |  | R | 22 | 64.44 | 59.09 | 5’-GCACACCAAGAACAGCTGCTCC-3’ |
| 1. 4 | 5'-diphospho-2-C-methyl-D-erythritol kinase | ec, 2.7.1.148 | unigene_3851 | F | 23 | 63.97 | 52.17 | 5’-ACCAATGTTCCAGGTGTCCCAGT-3’ |
|  |  |  |  | R | 22 | 65.83 | 59.09 | 5’-ACTGCTTCCACCACCGAGACCA-3’ |
| 1. 5 | Histidinol-phosphate transaminase | ec, 2.6.1.9 | unigene_18410 | F | 22 | 63.86 | 54.55 | 5’-TCGGGCAGTCTTGGAACCTTGA-3’ |
|  |  |  |  | R | 22 | 61.20 | 50.00 | 5’-ACATACTTGCTGGATGTGGTGC-3’ |
| 1. 6 | Aspartate aminotransferase | ec, 2.6.1.1 | unigene_2985 | F | 21 | 64.31 | 57.14 | 5’-TCCCCACGAAGAACAGCAGCA-3’ |
|  |  |  |  | R | 22 | 64.32 | 59.09 | 5’-CGGGGCTGGGATCCTTGTTGTA-3’ |
| 1. 7 | 15-cis-phytoene synthase | ec, 2.5.1.32 | unigene_29008 | F | 21 | 63.48 | 57.14 | 5’-TTCTGTCAGACCTGCTTGCGC -3’ |
|  |  |  |  | R | 22 | 64.06 | 59.09 | 5’-CAGTTCCTGTCATGGGCATCGC-3’ |
| 1. 8 | [(2E,6E)-farnesyl-diphosphate specific] synthase | ec, 2.5.1.31 | unigene_7184 | F | 21 | 64.05 | 57.14 | 5’-AGTGAGGTTGGCGGCTCAGAA-3’ |
|  |  |  |  | R | 22 | 64.12 | 50.00 | 5’-TGCACACCGTTATTTGCAGCGT-3’ |
| 1. 9 | Squalene synthase | ec, 2.5.1.21 | unigene_6679 | F | 22 | 65.43 | 59.09 | 5’-AGGTGAAGGTGCCCATCCTCCA-3’ |
|  |  |  |  | R | 22 | 64.96 | 59.09 | 5’-CCATTCCTGCTCCCATTCGCCT-3’ |
| 1. 11 | 1-deoxy-D-xylulose-5-phosphate synthase | ec, 2.2.1.7 | unigene_891 | F | 21 | 65.48 | 57.14 | 5’-ATGGACACGGCGTTTCTGCGT-3’ |
|  |  |  |  | R | 22 | 65.11 | 59.09 | 5’-GCCCCTTGATGGTGAAGCTGGT-3’ |
| 1. 12 | S-isoprenylcysteine O-methyltransferase | ec, 2.1.1.100 | unigene_9003 | F | 22 | 65.10 | 59.09 | 5’-TGCCTCTGGTCTAAAAGCGCCC -3’ |
|  |  |  |  | R | 21 | 64.59 | 57.14 | 5’-AAACTGCCACGCCTGTCTCCA-3’ |
| 1. 13 | (E)-4-hydroxy-3-methylbut-2-enyl-diphosphate synthase | ec, 1.17.7.1 | unigene_20792 | F | 22 | 63.96 | 54.55 | 5’-TCTGCCAGGTGTCTCGATTGCT-3’ |
|  |  |  |  | R | 22 | 64.93 | 59.09 | 5’-CGGCCATGGTCCTTGATCAGCT-3’ |
| 1. 14 | 4-hydroxy-3-methylbut-2-enyl diphosphate reductase | ec, 1.17.1.2 | unigene_18093 | F | 21 | 63.18 | 57.14 | 5’-GCTCAAGGAGAACGGCAACCA-3’ |
|  |  |  |  | R | 21 | 64.21 | 57.14 | 5’-TGTTGACGGTGGGGTTGTGGA-3’ |
| 1. 15 | Squalene monooxygenase | ec, 1.14.13.132 | unigene_1811 | F | 21 | 64.96 | 57.14 | 5’-ATGCCCAGCGTGTCCTTGGTT-3’ |
|  |  |  |  | R | 22 | 65.05 | 59.09 | 5’-GGACATTGGGCAAAGACGCAGC-3’ |
| 1. 16 | 1-deoxy-D-xylulose 5-phosphate reductoisomerase | ec, 1.1.1.267 | unigene_2598 | F | 21 | 63.75 | 57.14 | 5’-TGGTCCTTTCGTGCTTCCCCT-3’ |
|  |  |  |  | R | 22 | 64.76 | 54.55 | 5’-TGGCCAATCCCTGAAAGCACCA-3’ |
| 1. 17 | HMG-CoA reductase | ec, 1.1.1.34 | unigene_3740 | F | 20 | 64.70 | 60.00 | 5’-TGCAGCACAGGGGATGCCAT-3’ |
|  |  |  |  | R | 21 | 63.89 | 57.14 | 5’-TCCCACGGCCATCAATCCAG-3’ |
| 1. 18 | Geranylgeranyl pyrophosphate synthase | ec, 2.5.1.29 | unigene_9252 | F | 22 | 63.95 | 59.09 | 5’-ACTCACGTCATCTCCCACACCC-3’ |
|  |  |  |  | R | 22 | 64.03 | 59.09 | 5’-GCCATCTCGCCGAACTTCTTGG-3’ |
| 1. 19 | Acetyl-CoA carboxylase | ec, 6.4.1.2 | unigene_2 | F | 23 | 64.84 | 56.52 | 5’-GCCCAGTTACTGTTGCTCCTCGT-3’ |
|  |  |  |  | R | 22 | 63.17 | 54.55 | 5’-TCGGTTACTGGATGCTCGACCT-3’ |
| 1. 20 | Isopentenyl-diphosphate Delta-isomerase | ec, 5.3.3.2 | unigene_10518 | F | 22 | 63.29 | 50.00 | 5’-TGGAAAATGGGGCGAACACGAA-3’ |
|  |  |  |  | R | 21 | 63.98 | 57.14 | 5’-TCCACCACCAGCCTGAACCAA-3’ |
| 1. 21 | Mevalonate kinase | ec, 2.7.1.36 | unigene_3404 | F | 22 | 64.08 | 59.09 | 5’-CGCTAGGGGAGATCAGTGCCAA-3’ |
|  |  |  |  | R | 20 | 64.19 | 60.00 | 5’-AGGTCACCACTGCCTTGCCA-3’ |

**
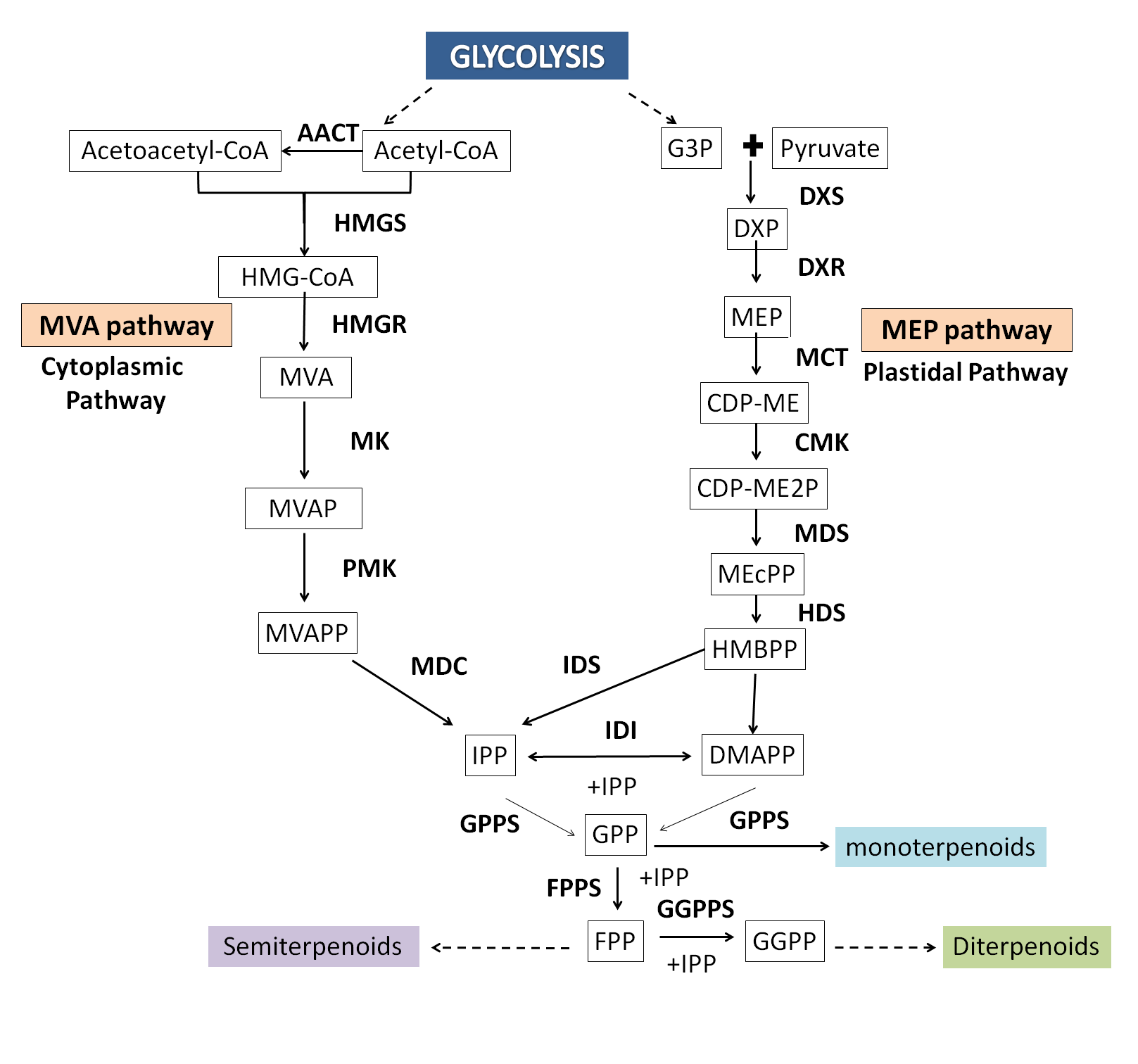
Figure S1|**. Terpenoid backbone synthesis pathway. It compise both MEP and MVA pathway. Pathway Mevalonate (MVA) cytoplasmic in left and pathway 1-deoxyxylulose-5-phosphate (DXP) chloroplastic in right. ***Enzymes involved in the pathway***: AACT, acetoacetyl-coenzyme A (CoA) thiolase; HMGS, HMG-CoA synthase; HMGR, 3-hydroxy-3-methylglutaryl coenzyme A (HMG-CoA) reductase; MK, mevalonate kinase; MPK, mevalonate-5-phosphate kinase; MDC, mevalonate diphosphate decarboxylase; DXS, 1-deoxy-D-xylulose 5-phosphate synthase; DXR, 1- deoxy-D-xylulose 5-phosphate reductoisomerase; MCT, 2-C-methyl-D-erythritol 4-phosphaate cytidyl transferase; CMK, 4-(cytidine 5'-diphospho)-2-C-methyl-D-erythritol kinase; MDS, 2-C-methyl-D-erythritol 2,4-cyclodiphosphate synthase; HDS, 1-hydroxy-2-methyl-2-(E)-butenyl 4-diphosphate synthase; IDS, isopentenyl diphosphate/dimethylallyl diphosphate synthase; GPPS, geranyl diphosphate synthase; FPPS, farnesyl diphosphate synthase; GGPPS, geranylgeranyl diphosphate synthase; IDI, isopentenyl diphosphate isomerase; ***Substrates:*** HMG-CoA, 3S-hydroxy-3-methylglutaryl coenzyme A; MVA, 3R-Mevalonic acid; MVAP, Mevalonate acid 5-phosphate; MVAPP, Mevalonate acid pyrophosphate; DXP, 1-deoxy-D-xylulose 5-phosphate; MEP, 2-C-methyl-D-erythritol 4-phosphate; CDP-ME, 4-(cytidine 5'-diphospho)-2C-methyl-D-erythritol; CDP-ME2P, 4-(cytidine 5'-diphospho)-2C-methyl-D-erythritol 2-phosphate; MEcPP, 2C-methyl-D-erythritol 2,4-cyclodiphosphate; HMBPP, 1-hydroxy-2-methyl-2-(E)-butenyl 4-diphosphate; IPP, isopentenyl diphosphate; DMAPP, Dimethylallyl diphosphate; GPP, geranyl diphosphate; FPP, farnesyl diphosphate; GGPP, geranylgeranyl diphosphate.

**
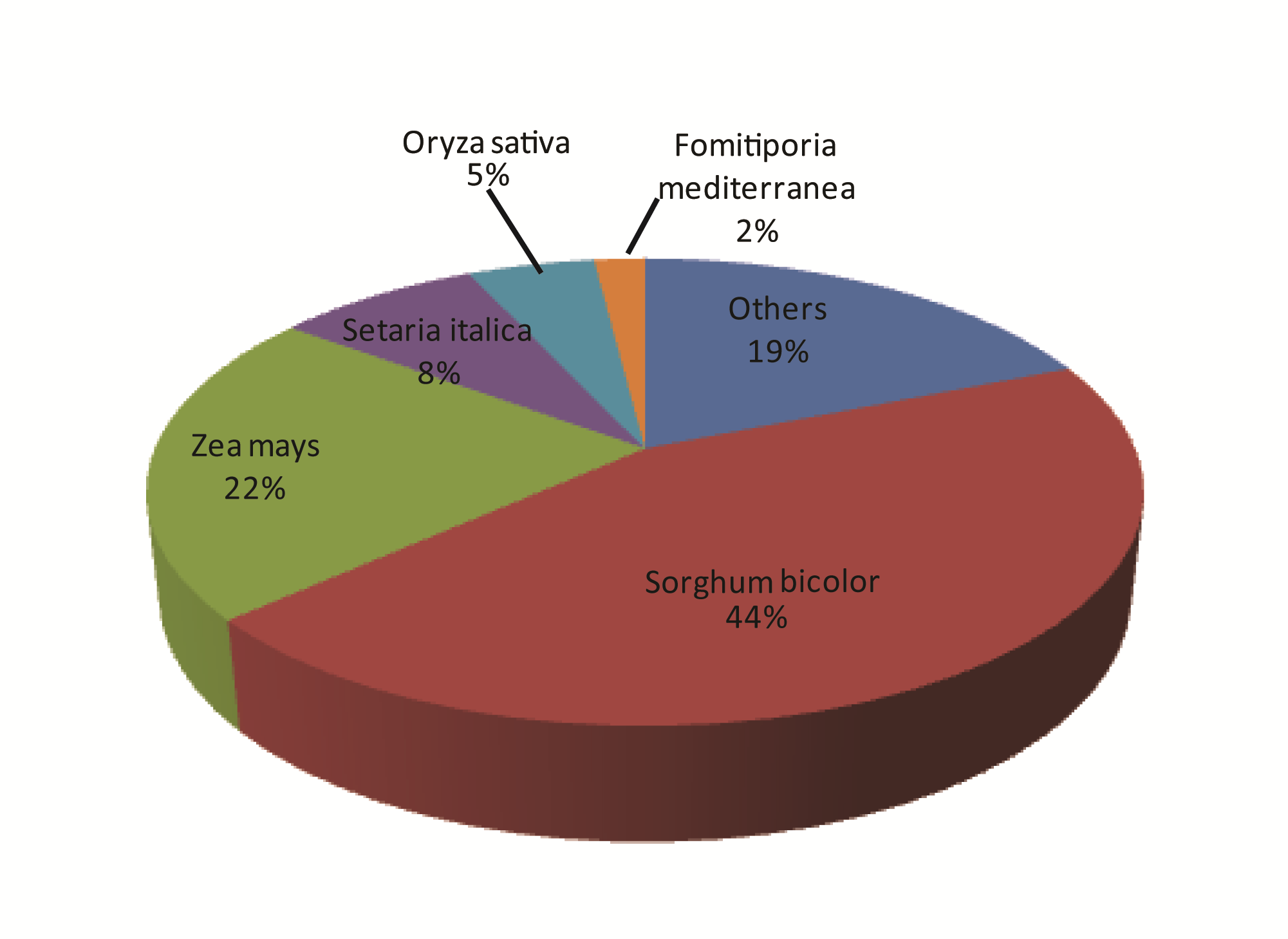
Figure S2|**. Functional Annotation of the unigenes


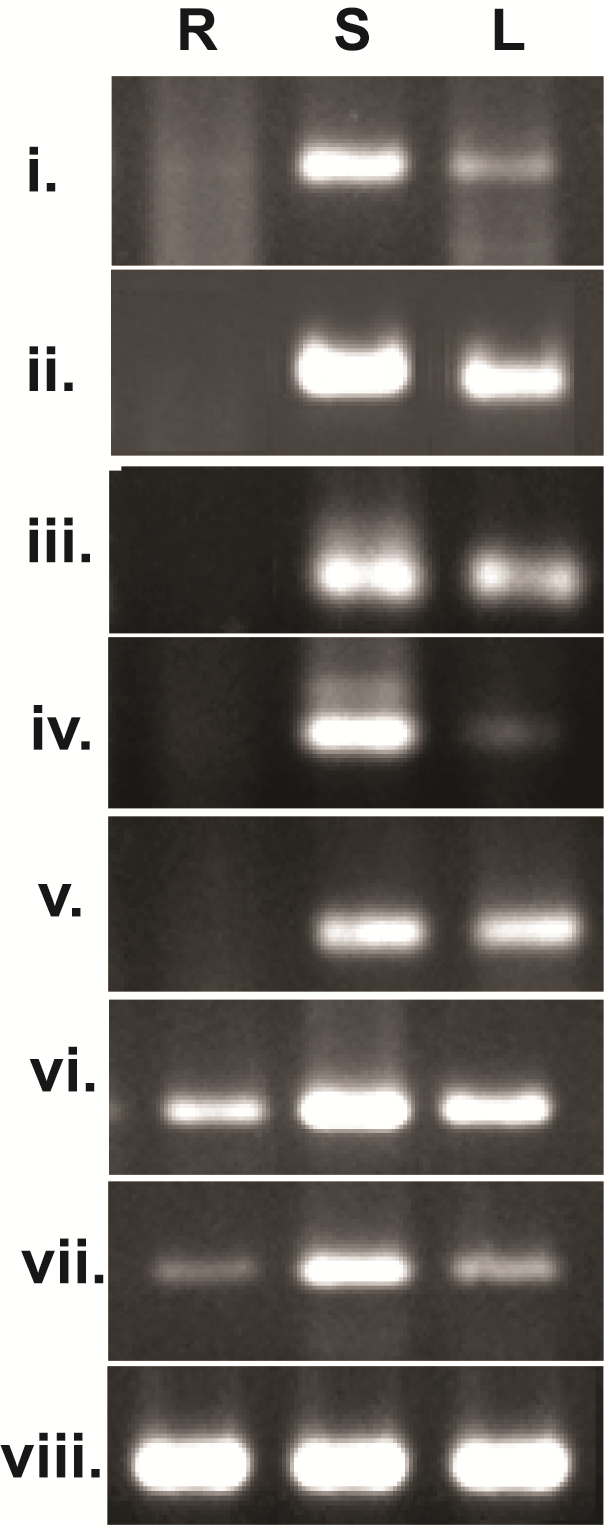


**Figure S3**| The differential expression study result of MEP pathway genes by RT PCR in root (R), leaf (L) and leaf sheath (S) of *C. winterianus* using GAPDH as internal control. i. 1-deoxy-D-xylulose 5-phosphate synthase, ii. 1-deoxy-D-xylulose5-phosphate reductoisomerase, iii. 4-phosphate cytidylyl transferase, iv. 5'-diphospho-2-C-methyl-D-erythritol kinase, v. 2,4-cyclodiphosphate synthase, vi. (E)-4-hydroxy-3-methylbut-2-enyl-diphosphate synthase, vii. 4-hydroxy-3-methylbut-2-enyl diphosphate reductase and viii. GAPDH.


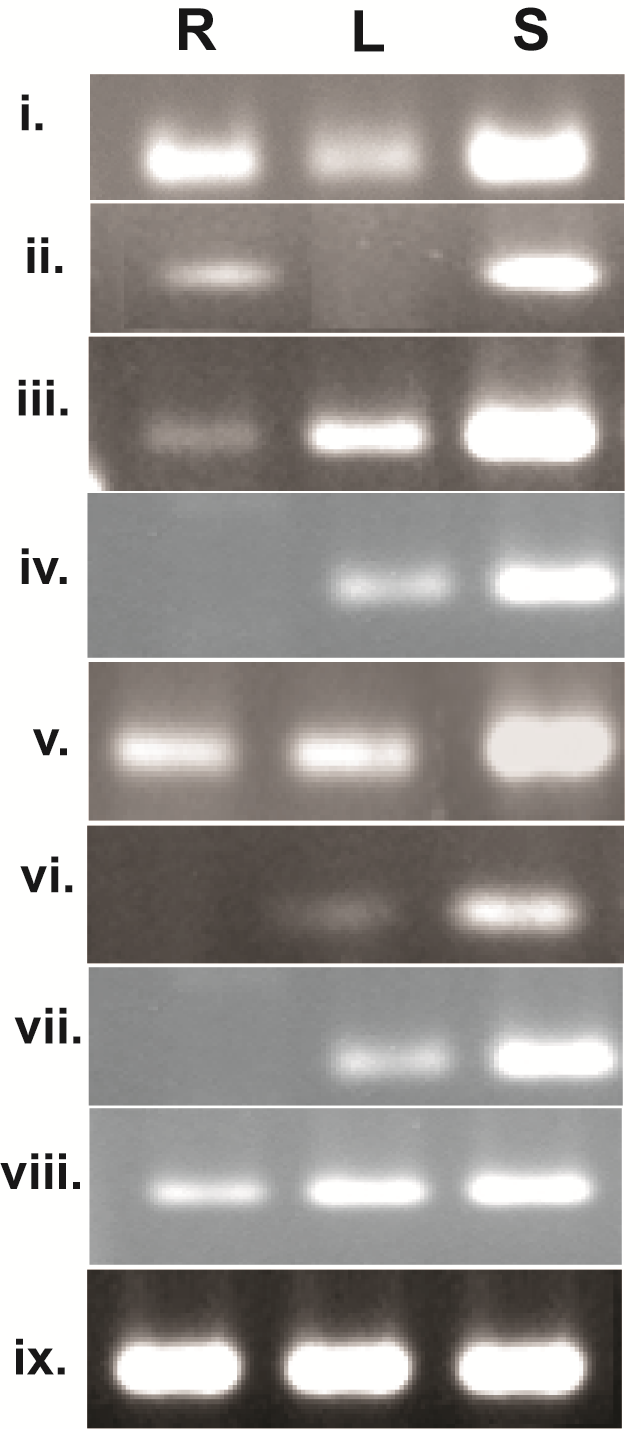


**Figure. S4|** Differential expression study result of four Mevalonate pathway genes by A. RT and B. qRT PCR in root (R), leaf (L) and leaf sheath (S) tissue of *C. winterianus* using GAPDH as internal control .i. HMG-CoA reductase, ii. Mevalonate kinase, iii. Diphosphomevalonate decarboxylase, iv. hydroxymethylglutaryl-CoA synthase, v. Geranylgeranyl pyrophosphate synthase, vi. (2E,6E)-farnesyl-diphosphate synthase, vii. S-isoprenyl cysteine O-methyl transferase, viii. Isopentenyl-diphosphate Delta-isomerase and ix. GAPDH.


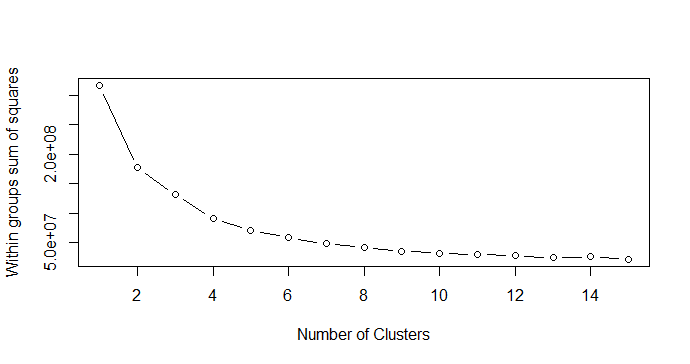


**Figure S5|**.Optimal K with WSS (Within groups Sum of Squares)
